# Machine learning-based nomogram for non-suicidal self-injury among depressed adolescents: a multicentre study

**DOI:** 10.3389/fpsyt.2026.1787248

**Published:** 2026-07-17

**Authors:** Lan Hong, Jianuo Shi, Qianjin Lou, Ying Yao, Tianshu Peng, Zhen Xu, Jinwei Gai, Zhaoxuan Liu, Siyu Tong, Tiansheng Zheng, Dongwu Xu, Ke Zhao

**Affiliations:** 1 Lishui Second People’s Hospital Affiliated to Wenzhou Medical University, Lishui, China; 2 School of Mental Health, Wenzhou Medical University, Wenzhou, China; 3 Second Clinical Medical College, Wenzhou Medical University, Wenzhou, China; 4 Carnegie Mellon University, Pittsburgh, PA, United States; 5 Zhejiang Provincial Clinical Research Center for Mental Disorders, The Affiliated Wenzhou Kangning Hospital, Wenzhou Medical University, Wenzhou, China

**Keywords:** cross-sectional study, depression, nomogram, NSSI, random forest

## Abstract

**Background:**

Non-suicidal self-injury (NSSI) is a major clinical concern among adolescents with depression, but reliable tools for individualized risk assessment are limited. This study aimed to develop and validate a clinically applicable nomogram for estimating the probability of NSSI in this population.

**Methods:**

Data were obtained from a nationwide multicenter cohort of 2, 343 adolescents with depression recruited from 14 hospitals in China. Participants were randomly divided into training and validation sets. Variables associated with NSSI were selected by utilizing Random Forest integrated with SHAP values combined with logistic regression. Model performance was assessed using the area under the receiver operating characteristic curve (AUC), the Hosmer–Lemeshow test, calibration curves, and decision curve analysis (DCA).

**Results:**

Eight variables were identified, including depression score, sleep medication use, difficulty identifying feelings, age, perceived family support, female, hallucination and externally oriented thinking. The nomogram performed well in both the training and validation cohorts, as evidenced by AUC values of 0.754 (95% CI: 0.726-0.781) and 0.748 (95% CI: 0.707-0.789), together with the calibration curves and DCA.

**Conclusion:**

A nomogram integrating eight clinical and psychosocial variables was developed and validated to estimate NSSI risk among adolescents with depression. This tool may help clinicians estimate the current probability of NSSI and support further psychosocial assessment and individualized clinical management.

## Introduction

Non-suicidal self-injury (NSSI) refers to the deliberate and self-inflicted damage to one’s body without suicidal intent and for purposes not culturally sanctioned (1). This behavior is closely associated with psychopathology. Among patients who engage in self-injury, more than 80% have comorbid mental disorders. The most common conditions include major depressive disorder (MDD), anxiety disorders, and alcohol use disorder (2). Rates of NSSI are notably elevated among younger people, including adolescents (17–27%) (3–5) and young adults (13–18%) (4), especially among psychiatric inpatients (12–80%) (6). An estimated 70% of adolescent psychiatric inpatients engage in NSSI (7). Despite its clinical significance, most individuals with NSSI do not receive adequate psychosocial assessment or intervention (8). NSSI is strongly associated with long-term suicide risk (9), with approximately 0.7% of adolescents dying by suicide within one year following their first NSSI episode (10). Compared with a history of suicide attempts (SAs), NSSI is a stronger predictor of subsequent suicide-related behaviors (11). In recognition of its clinical relevance, the DSM-5 has included NSSI as a condition for further study (12).

Although previous studies have examined factors associated with NSSI from various perspectives, a single risk-related factor or theoretical framework is often insufficient to fully explain its risk. This may be especially true for adolescents with depression, among whom NSSI often co-occurs with emotional distress, cognitive processing difficulties, interpersonal stress, and environmental stressors. Therefore, the occurrence of NSSI is characterized by multifactorial and heterogeneous risk patterns. Compared with focusing on a single category of variables, integrating risk factors from multiple domains into one modeling framework may help identify key warning indicators and improve the model’s clinical utility for screening.

In this study, candidate variables for NSSI were selected based on previous literature and the biopsychosocial framework (13, 14). Biological and demographic factors, including sex and age (1, 15), were included to reflect basic individual susceptibility to NSSI. Psychological variables, including depression (16, 17), anxiety (18), alexithymia (19, 20), sleep problems (21), and related emotional and cognitive features, were included to assess internal psychological distress and difficulties in emotion processing. Social factors, including childhood trauma (16, 22, 23), peer relationships (24), school environment (25), and social support (26), were included to capture the roles of external contexts and interpersonal relationships in NSSI risk. Additionally, within the psychotic experience spectrum, auditory hallucinations (AHs) are an especially potent risk factor for self-injurious behavior (SIB) and suicidality (27); these associations have been identified even where other PE subtypes demonstrated no such association (28). Moreover, AH items have been shown to demonstrate the greatest validity among adolescent PE screeners (29). Integrating these variables within the biopsychosocial framework helps maintain theoretical clarity while improving the model’s ability to capture multidimensional risk information, thereby providing a basis for developing more targeted tools for NSSI risk assessment and intervention.

Previous research on NSSI has largely relied on traditional statistical methods with limited variable selection, which may result in substantial information loss and models with limited ecological validity (30). Given the complexity of NSSI, integrating machine learning (ML) algorithms may help identify key variables and capture complex patterns among multiple factors. Prior studies have demonstrated that ML offers advantages in modeling complex relationships among variables and improving model performance (31). It is particularly useful when multiple predictors are involved, especially in the presence of nonlinear patterns and interaction effects that are difficult to conceptualize theoretically and to model using conventional approaches (32). ML has been widely applied in mental health research. A previous study suggested that, compared with traditional model-based statistical methods, ML approaches show higher sensitivity and accuracy in identifying suicidal behaviors (33). For instance, previous studies have used penalized logistic regression within ML frameworks to construct classification models for distinguishing suicidal thoughts and behaviors in children (34). Other researchers have applied ML algorithms to examine interrelationships among relevant factors and further select models for identifying self-injurious behaviors based on these findings (35, 36).

Building on prior research on self-injury risk identification, this study focused on adolescents with depression and the multidimensional risk profile of NSSI. We first included candidate variables across biological and demographic factors, psychological symptoms, emotional and cognitive features, and social environmental factors. Multiple ML algorithms were then used to compare model performance, and SHAP analysis was further applied to identify key variables that made strong contributions to model output. Next, based on the important variables identified by machine learning, we built a parametric logistic regression model and developed a nomogram to translate complex model results into a more intuitive and interpretable tool for individual-level risk assessment. Compared with simply reporting the performance of machine learning models, this analytic process may broaden variable selection and improve model performance, while also enhancing the readability and practical value of the results in clinical assessment settings. This study aims to provide quantitative evidence for NSSI risk assessment among adolescents with depression and to inform clinical screening and individual-level assessment and prevention planning.

## Methods

### Study design and sample

Participants were recruited from 14 collaborative research centers nationwide between January 2021 to June 2022. The study protocol has been approved by the Institutional Review Board, and all relevant procedures comply with the requirements of the Declaration of Helsinki. At each center, uniformly trained researchers, together with clinicians and school mental health professionals, screened adolescents based on predefined inclusion and exclusion criteria. Eligible and voluntary participants were enrolled using multicenter convenience sampling. Prior to the survey, all adolescents and their guardians were fully informed of the study objectives, procedures, confidentiality rules and the principle of voluntary participation, after which written informed consent was collected. A total of 481 participants were excluded during data cleaning, mainly due to failure to meet the inclusion criteria or invalid questionnaires, such as extremely short completion time and identical answers to all items. The final analytical dataset comprised 2, 343 participants with complete data for all variables in this study.

#### Inclusion criteria

- participants met the diagnostic criteria for depressive episodes or depressive episodes associated with bipolar disorder as specified in the Diagnostic and Statistical Manual of Mental Disorders, Fifth Edition (DSM-5);
- aged 12–18 years;
- before participating in this study, all participants were provided with a detailed explanation of the informed consent by research administrators who had received unified training. Participants and their legal guardians fully understood the relevant information about the study and voluntarily signed the written informed consent form. Each participant was assigned a unique ID for identification, and their personal information was anonymized before being entered into the data system.

#### Exclusion criteria

- participants with severe physical diseases, infectious diseases, or complications of immune system diseases;
- participants with a history of traumatic brain injury, epilepsy, or other known severe neurological diseases or organic brain diseases;
- participants with a history of severe mental disorders such as schizophrenia or intellectual disability.

After obtaining informed consent, all participants underwent a comprehensive interview assessment conducted by two experienced psychiatrists. Prior to the initiation of the study, all members of the research team at each center received unified training.

### Measures

#### Sociodemographic variables

Basic information of participants was collected using a self-designed general demographic information questionnaire, which mainly included: age, gender, education years, physical disease (yes, no), location (rural, city), parents’ educational background, family history of mental illness (yes, no), and annual family income.

#### NSSI

The Chinese Version of the Functional Assessment of Self-Mutilation (FASM) was used to assess participants’ NSSI (37). The scale inquired about their engagement NSSI over the past 12 months, such as cutting or carving the skin. Participants who engaged in any type of NSSI in the past 12 months were classified as having recent NSSI. The Cronbach’s alpha value of FASM was 0.86.

#### Depression

The Patient Health Questionnaire-9 (PHQ-9), which consists of 9 items, uses a Likert 4-point scale ranging from 0 (very little time) to 3(most time) to assess the degree of depressive symptoms. Studies have shown that PHQ-9 can be used to assess the severity of depression in adolescents (38). The Cronbach’s alpha value of PHQ-9 was 0.90.

#### Anxiety

The Generalized Anxiety Disorder-7 (GAD-7), which is composed of 7 items, adopts a Likert 4-point scale ranging from 0 (not at all) to 3 (nearly every day) to evaluate the severity of generalized anxiety symptoms. Studies have demonstrated that GAD-7 is applicable for assessing anxiety symptom severity in adolescents (39). The Cronbach’s alpha value of GAD-7 in this study was 0.92.

#### Sleep quality

The Pittsburgh Sleep Quality Index (PSQI) was used to assess the sleep quality of adolescents over the past month (40). This scale consists of 7 dimensions including subjective sleep quality, sleep latency, sleep duration, sleep efficiency, sleep disturbances, use of sleeping medication, and daytime dysfunction. Each dimension is scored on a scale of 0 to 3. The Cronbach’s α coefficient of this scale was 0.70.

#### Alexithymia

The Toronto Alexithymia Scale (TAS) is a 20-item self-report instrument, with items rated on a 5-point Likert scale (1 = strongly disagree to 5 = strongly agree). It includes three core dimensions: Difficulty Identifying Feelings (DIF), Difficulty Describing Feelings (DDF), and Externally-Oriented Thinking (EOT) (41). Its overall Cronbach’s α was 0.81, with subscale coefficients of 0.89 for DIF, 0.71 for DDF, and 0.72 for EOT.

#### Peer victimization

Peer victimization was assessed using the 16-item Multidimensional Peer Victimization Scale (MPVS) (42). The scale covers four dimensions: physical victimization (PV), verbal victimization (VV), social manipulation (SM), and attacks on property (AP), and demonstrates good reliability and validity in younger populations. Its overall Cronbach’s α was 0.92, with subscale coefficients of 0.82 for physical victimization, 0.85 for verbal victimization, 0.90 for social manipulation, and 0.85 for attacks on property.

#### Perceived social support

The 12-item Multidimensional Scale of Perceived Social Support (MSPSS) assesses perceived social support adequacy from three domains: family, friends, and significant others (43). Using a 7-point Likert scale (1 = strongly disagree; 7 = strongly agree). Its overall Cronbach’s α was 0.92, with subscale coefficients of 0.78 (family support), 0.80 (friend support), and 0.81 (significant other support).

#### Childhood trauma

Childhood trauma was assessed via the CTQ, which covers five dimensions: emotional abuse, physical abuse, sexual abuse, emotional neglect, and physical neglect (44). The scale’s overall Cronbach’s α was 0.85, with subscale coefficients ranging from 0.74 (emotional abuse) to 0.86 (sexual abuse), showing acceptable reliability.

### Statistical analyses

#### Data preprocessing

All data analyses were performed using R (version 4.5.2) (45). Before model development, the raw data were cleaned, encoded, and split into training and validation cohorts at a 7:3 ratio. Detailed variable definitions and coding schemes are provided in **Supplementary Table 3**. All preprocessing steps were conducted within the training cohort and then applied to the validation cohort to avoid information leakage. Continuous variables were standardized to a mean of 0 and a standard deviation of 1 for scale-sensitive models, including LASSO, SVM, and KNN, while categorical variables were dummy-coded for these models. No standardization was applied to tree-based models, including decision tree, random forest, XGBoost, and LightGBM, and Naive Bayes was fitted without standardization.

#### Model development and evaluation

First, NSSI was treated as the outcome variable (0 = no NSSI; 1 = NSSI), and sociodemographic and psychosocial factors were included as candidate variables. Eight machine-learning algorithms were developed and compared for NSSI prediction. Each model’s performance was evaluated in the validation cohort using multiple complementary metrics, including accuracy, AUROC, sensitivity, positive predictive value (PPV), and Brier Score (36). Hyperparameters were tuned within the training cohort using model-specific procedures, including grid/random search, cross-validation, out-of-bag estimation, or internal validation, with AUROC as the primary optimization criterion. Class imbalance was handled using model-specific strategies where applicable. For example, sampling schemes were applied for the random forest model, whereas class weights were adjusted for XGBoost and LightGBM. Classification thresholds were estimated exclusively from training-set predictions, mainly according to the Youden index, and then fixed for validation; the validation cohort was kept unchanged to preserve the original outcome distribution. Detailed hyperparameter search ranges and final selected values are provided in **Supplementary Table 4**. Considering the overall performance profile across these metrics, the best-performing model was selected for subsequent analyses. Subgroup validation was then conducted for the selected model in the validation cohort, stratified by sex (male/female) and age group (<15years/≥15 years). SHAP (SHapley Additive exPlanations) analysis was implemented for the overall optimal model. SHAP values were used to assess the relative importance of each predictor in the model, rank predictors according to mean absolute SHAP values, and visualize how predictor values were associated with the predicted probability of NSSI.

#### Nomogram construction and performance evaluation

The top 20 variables identified by the machine learning model were further examined using multivariable logistic regression to support model interpretation and nomogram construction. A nomogram was then developed based on the final selected variables. Discrimination, calibration, and clinical utility were evaluated in the training and validation sets using ROC curves (46), calibration curves, and decision curve analysis (DCA) (47) respectively.

## Results

### Baseline characteristics of NSSI

Among the 2, 343 participants, the mean age was 15.0 years (SD = 1.65). Most participants resided in urban areas, and 1, 826 (77.9%) were female. Detailed demographic and clinical characteristics are presented in **Table 1**. The cohort was randomly divided into a training set (n = 1, 641) and a validation set (n = 702). No statistically significant differences were observed between the two sets (*p* > 0.05), as detailed in **Supplementary Table 2**. The results of multicollinearity diagnostics indicated no severe multicollinearity among the variables, with all VIF below 10 (**Supplementary Table 1**).

**Table 1:** Baseline characteristics of all the participants included.

Variable	Overall	No-NSSI (n=561)	NSSI (n = 1782)
Family history of mental disorders (FHMD) (n, %)			
No	2121 (90.5)	512 (91.3)	1609 (90.3)
Yes	222 (9.5)	49 (8.7)	173 (9.7)
Age (mean, SD)	14.99 (1.65)	15.42 (1.60)	14.85 (1.64)
Gender (n, %)			
Boy	517 (22.1)	199 (35.5)	318 (17.8)
Girl	1826 (77.9)	362 (64.5)	1464 (82.2)
Education years (EY) (mean, SD)	9.16 (1.74)	9.55 (1.72)	9.04 (1.73)
Location (n, %)			
Rural	763 (32.6)	165 (29.4)	598 (33.6)
City	1580 (67.4)	396 (70.6)	1184 (66.4)
Annual household income (AHI) (n, %)			
≤ 20, 000	345 (14.7)	74 (13.2)	271 (15.2)
20, 000 - 50, 000	269 (11.5)	58 (10.3)	211 (11.8)
50, 000 - 100, 000	408 (17.4)	87 (15.5)	321 (18.0)
100, 000 - 500, 000	562 (24.0)	149 (26.6)	413 (23.2)
> 500, 000	759 (32.4)	193 (34.4)	566 (31.8)
Education level - Father (EF) (n, %)			
≤6 years	301 (12.8)	68 (12.1)	233 (13.1)
7–9 years	882 (37.6)	195 (34.8)	687 (38.6)
10–12 years	527 (22.5)	134 (23.9)	393 (22.1)
≥13 years	633 (27.0)	164 (29.2)	469 (26.3)
Education level - Mather (EM) (n, %)			
≤6 years	532 (22.7)	124 (22.1)	408 (22.9)
7–9 years	809 (34.5)	189 (33.7)	620 (34.8)
10–12 years	453 (19.3)	110 (19.6)	343 (19.2)
≥13 years	549 (23.4)	138 (24.6)	411 (23.1)
Physical disease (PD) (n, %)			
No	2278 (97.2)	546 (97.3)	1732 (97.2)
Yes	65 (2.8)	15 (2.7)	50 (2.8)
Hallucination (n, %)			
None	1471 (62.8)	439 (78.3)	1032 (57.9)
Possible	679 (29.0)	100 (17.8)	579 (32.5)
Confirmed	193 (8.2)	22 (3.9)	171 (9.6)
Delusion (n, %)			
None	1472 (62.8)	405 (72.2)	1067 (59.9)
Possible	485 (20.7)	100 (17.8)	385 (21.6)
Confirmed	386 (16.5)	56 (10.0)	330 (18.5)
MPSS (mean, SD)			
Support from Family(SFA)	15.10 (6.15)	17.28 (6.20)	14.42 (6.00)
Support from Friends(SFR)	16.10 (6.82)	17.44 (6.54)	15.67 (6.85)
Support from Significant others(SSO)	15.87 (7.12)	16.69 (6.95)	15.61 (7.16)
TAS (mean, SD)			
Difficulty Identifying Feelings (DIF)	26.31 (6.04)	23.60 (6.49)	27.16 (5.63)
Difficulty Describing Feelings (DDF)	18.26 (3.52)	17.03 (3.68)	18.65 (3.38)
Externally Oriented Thinking (EOT)	23.28 (3.75)	22.21 (3.85)	23.61 (3.66)
CTQ (mean, SD)			
Physical abuse(PB)	7.19 (3.49)	6.79 (3.07)	7.32 (3.61)
Physical neglect(PN)	10.36 (3.33)	9.64 (3.09)	10.58 (3.37)
Emotional neglect(EN)	16.02 (5.04)	14.50 (5.22)	16.50 (4.88)
Emotional abuse(EA)	11.13 (4.73)	9.56 (4.14)	11.62 (4.80)
Sexual abuse(SA)	5.88 (2.41)	5.65 (1.87)	5.96 (2.55)
Bully (mean, SD)			
Physical victimization(PV)	1.94 (2.57)	1.45 (2.320)	2.10 (2.623)
Social manipulation(SM)	3.42 (3.19)	2.45 (2.932)	3.73 (3.21)
Verbal victimization(VV)	3.65 (3.02)	2.66 (2.79)	3.96 (3.019)
Attacks on property(AP)	2.34 (2.69)	1.65 (2.305)	2.56 (2.76)
PSQI (mean, SD)			
Subjective sleep quality (SQ)	1.69 (0.82)	1.44 (0.84)	1.77 (0.84)
Sleep latency (SI)	1.89 (1.03)	1.59 (1.07)	1.98 (1.01)
Sleep duration (SD)	1.23 (1.07)	1.01 (1.02)	1.30 (1.07)
Habitual sleep efficiency (HSE)	0.97 (1.06)	0.79 (0.95)	1.03 (1.08)
Sleep disturbances (SDI)	1.51 (0.72)	1.27 (0.70)	1.59 (0.71)
Use of sleeping medication (USM)	1.07 (1.32)	0.65 (1.13)	1.21 (1.35)
Daytime dysfunction (DY)	2.46 (0.82)	2.20 (0.97)	2.54 (0.75)
PHQ (mean, SD)	16.86 (7.17)	13.33 (7.42)	17.97 (6.72)
GAD (mean, SD)	12.10 (6.25)	9.63 (6.31)	12.87 (6.02)

### Machine learning model performance and variable selection

Eight suitable machine learning algorithms were used to train the machine learning model. Overall, the models showed varying performance across different evaluation metrics (**Table 2**). The random forest achieved relatively balanced results, with an accuracy of 0.729, AUC of 0.755, sensitivity of 0.773, positive predictive value (PPV) of 0.857, and a Brier score of 0.163. In the validation set, the model showed TP = 413, TN = 99, FP = 69, and FN = 121, with a specificity of 0.589, NPV of 0.450, balanced accuracy of 0.681, and PR-AUC of 0.902 (**Supplementary Table 5**).

**Table 2:** Comparison of machine learning model performance for predicting NSSI.

Model	Accuracy	AUC	Sensitivity	PPV	Brier Score
Decision tree	0.660	0.749	0.629	0.891	0.193
Random Forest	0.729	0.755	0.773	0.857	0.163
XGBoost	0.682	0.759	0.680	0.875	0.199
LASSO	0.635	0.771	0.582	0.904	0.152
SVM	0.577	0.710	0.519	0.874	0.167
LightGBM	0.761	0.747	0.837	0.847	0.175
KNN	0.677	0.709	0.700	0.848	0.166
Naive Bayes	0.648	0.738	0.614	0.889	0.248

AUC, area under the curve; PPV, positive predictive value, Brier Score lower indicates better accuracy. XGBoost, eXtreme Gradient Boosting; LASSO, least absolute shrinkage and selection operator; SVM, support vector machine; LightGBM, light gradient boosting machine; KNN, k-nearest neighbors.

Subgroup validation of the random forest model was conducted in the validation cohort stratified by sex and age group **Supplementary Table 8**. AUC values across subgroups were 0.787 (males), 0.726 (females), 0.794 (<15 years) and 0.717 (≥15 years), indicating satisfactory discrimination. Sensitivity remained high across all subgroups, ranging from 0.899 to 0.982. The most favorable overall performance was observed in aged <15 years, while the female subgroup also showed good classification performance.

**Figure 1:**
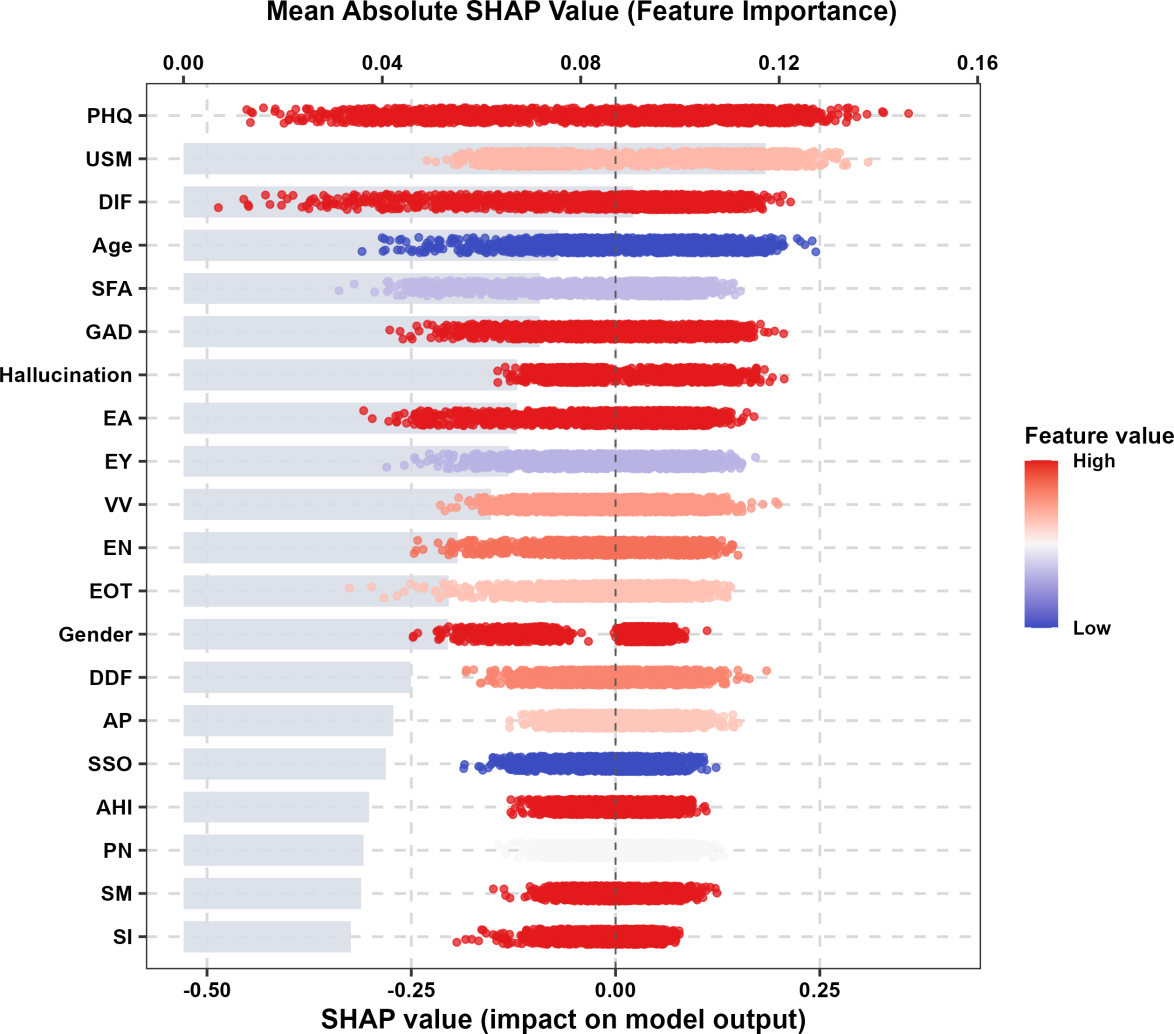
Variable importance and SHAP summary plots of the random forest model. The SHAP summary plot illustrates the contribution of each feature in the Random Forest model. A positive SHAP value for a given feature indicates an increased likelihood of NSSI, whereas a negative value indicates a lower likelihood. Each point represents one participant’s value for a given feature. The color of the points reflects the magnitude of the feature value: red represents a high feature value, whereas blue represents a low feature value.

Next, based on the mean absolute SHAP values, the top 20 key influencing factors were identified and ranked according to their SHAP values, as illustrated in **Figure 1**. Top 5 NSSI variables (mean |SHAP value|): depression (0.153), sleep medication use (0.117), difficulty identifying emotions (0.091), age (0.078) and family support (0.072).

**Figure 2:**
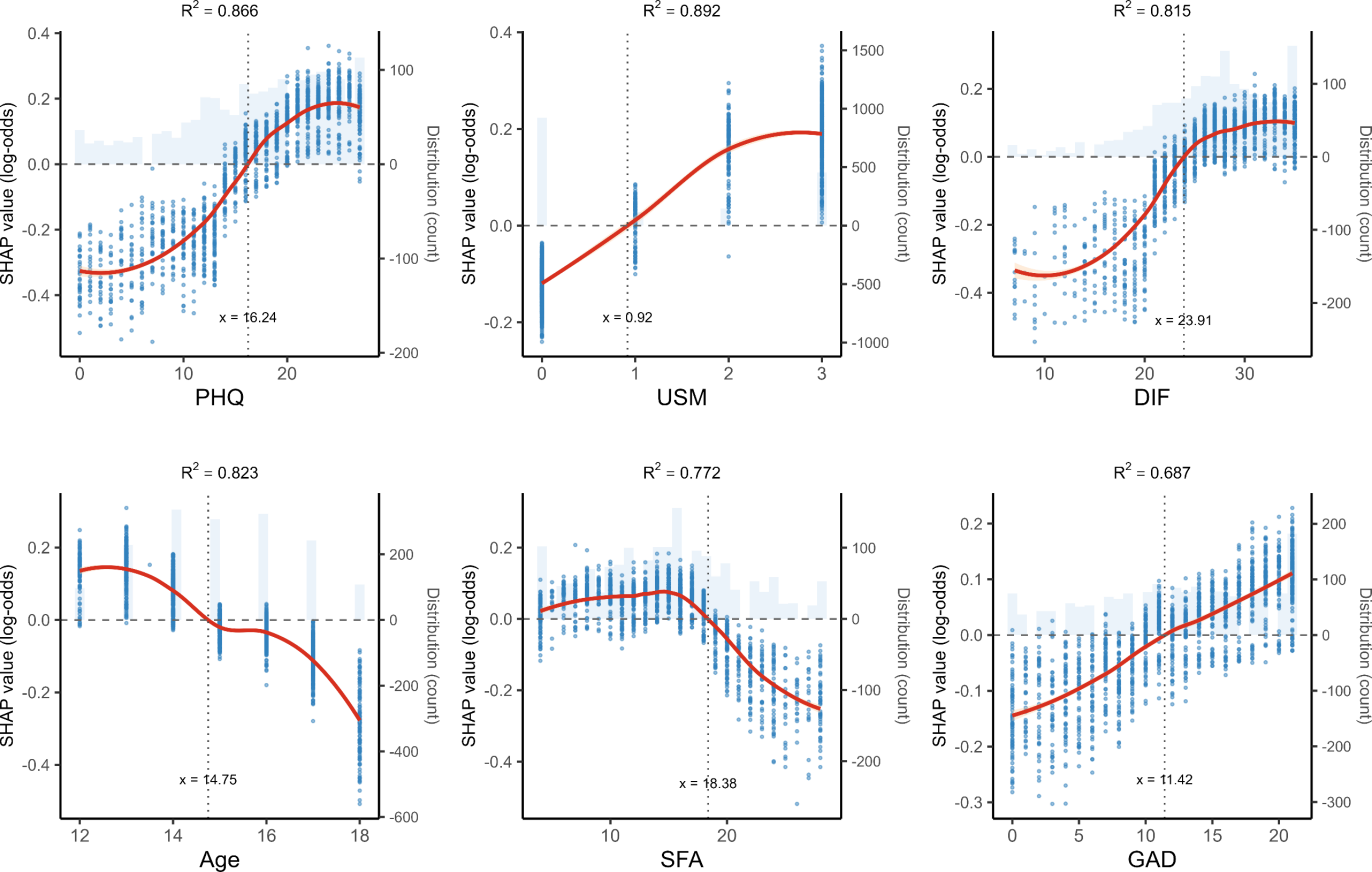
SHAP dependence plots of 6 features for NSSI. The y-axis represents the SHAP value for each feature, while the x-axis represents the feature values. Each plot includes sample data, a fitted line (with R² value), and a 95% confidence interval (CI), illustrating the relationship between the feature and NSSI. The trend line is generated using LOESS (Locally Weighted Regression) to describe the average marginal effect of each feature; the orange shaded area denotes the 95% confidence interval of the trend line. The dashed line represents the inflection point between SHAP values and NSSI risk. PHQ, Patient Health Questionnaire; USM, Use of sleeping medication; DIF, Difficulty Identifying Feelings; SFA, Support from Family; GAD, Generalised Anxiety Disorder scale.

Furthermore, **Figure 2** shows the direction and magnitude of the contributions of the top six key factors to NSSI classification. The decision boundary was 0, with SHAP values > 0 indicating a greater contribution toward classification as NSSI. The results indicate that these characteristics contributed to the model’s classification of adolescents as having NSSI: depression (PHQ-9 > 16.24), sleep medication use (USM > 0.92), difficulty identifying feelings (DIF > 23.91), age < 14.75 years, low family support (SFA < 18.38) and anxiety (GAD-7 > 11.42).

### Sensitivity analysis

A sensitivity analysis using the PHQ-8, excluding the PHQ-9 self-harm item (Item 9), showed results consistent with the main model. The PHQ-8 model had comparable validation performance, with an AUC of 0.775 and a Brier score of 0.179 (**Supplementary Table 6**). Overall, these differences were modest, supporting the robustness of the main findings. SHAP results were also stable, and depression severity remained an important contributor despite ranking lower. These findings suggest that the main results were not primarily driven by the PHQ-9 self-harm item (**Supplementary Table 7**).

### Construction of the nomogram-based risk identification model

**Figure 3:**
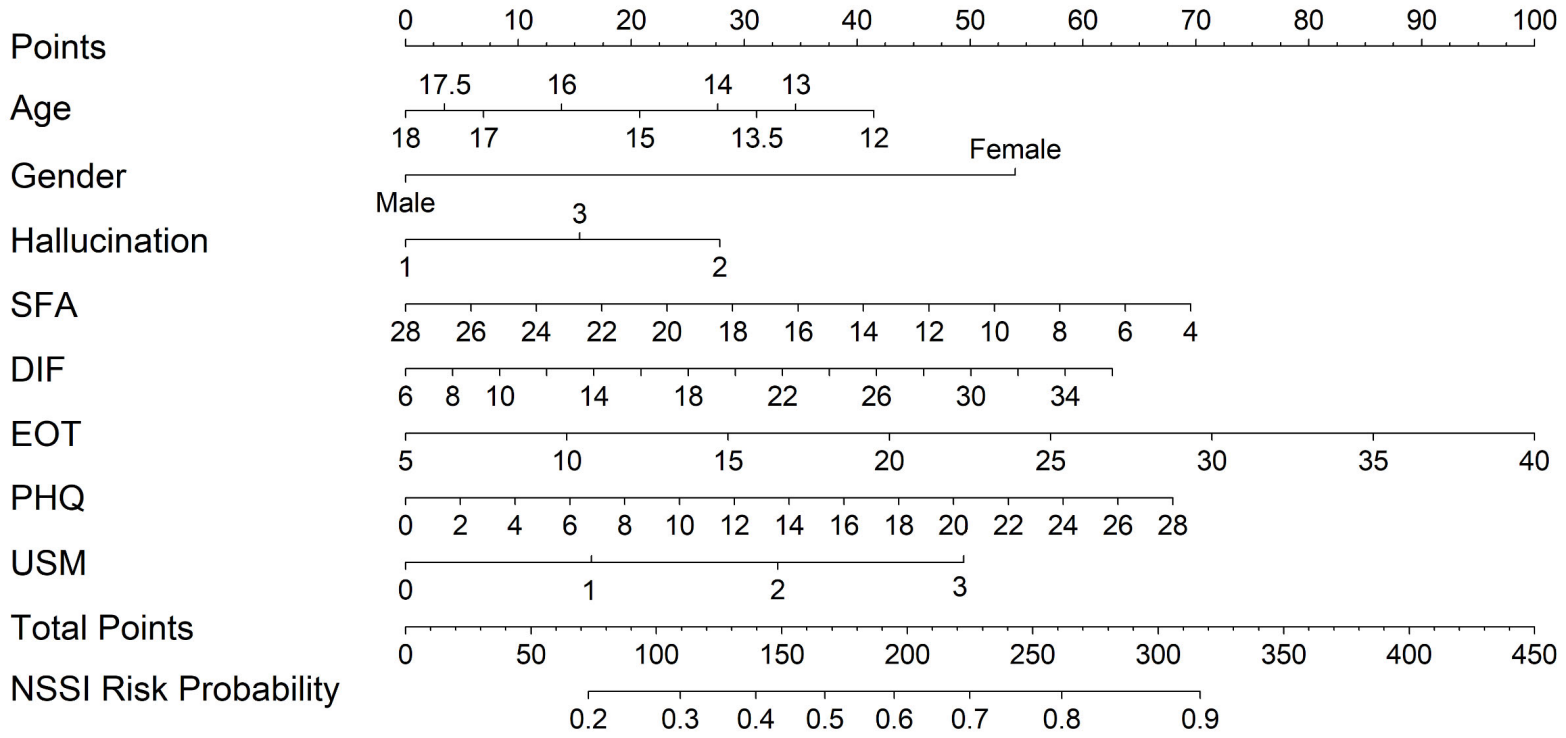
Nomogram for NSSI risk identification. USM, Use of sleeping medication; DIF, Difficulty Identifying Feelings; SFA, Support from Family; EOT, Externally Oriented Thinking; Hallucination (1=None; 2=Possible; 3=Confirmed).

NSSI status (0 = no NSSI; 1 = NSSI) was set as the dependent variable, and the variables identified by the random forest model were entered as independent variables in binary logistic regression analysis. Eight variables were significantly associated with NSSI: depression score (OR = 1.06, *p* = 0.001), sleep medication use (OR = 1.30, *p* < 0.001), difficulty identifying feelings (OR = 1.04, *p* = 0.015), age (OR = 0.85, *p* = 0.024), family support (OR = 0.95, *p* < 0.001), gender (boy, OR = 0.61, *p* = 0.001), hallucination (OR = 1.94, *p* < 0.001), and externally oriented thinking (OR = 1.05, *p* = 0.003). Details are presented in **Table 3**. Based on these variables, a nomogram-based model was developed for NSSI risk identification. The final nomogram included depression score, sleep medication use, difficulty identifying feelings, age, perceived family support, gender, hallucination, and externally oriented thinking style (**Figure 3**).

**Table 3:** Results of logistic regression.

Variables	OR	95% CI lower	95% CI upper	p
Gender				
Boy	0.61	0.45	0.81	0.001
Girl	Ref.			
Age	0.85	0.73	0.98	0.024
PHQ-9	1.06	1.02	1.10	0.001
GAD-7	0.98	0.95	1.02	0.32
USM	1.30	1.17	1.44	<0.001
DIF	1.04	1.01	1.08	0.015
SFA	0.95	0.93	0.97	<0.001
EY	1.02	.892	1.17	0.756
EOT	1.05	1.02	1.09	0.003
VV	1.06	0.99	1.12	0.075
EN	1.02	0.98	1.06	0.304
EA	1.02	0.98	1.06	0.344
DDF	1.04	1.01	1.08	0.015
Hallucination	1.94	1.62	2.34	<0.001
AP	1.01	0.95	1.07	0.73

USM, Use of sleeping medication; DIF, Difficulty Identifying Feelings; SFA, Support from Family; EY, Education years; EOT, Externally Oriented Thinking; VV, Verbal victimization; EN, Emotional neglect; EA, Emotional abuse; DDF, Difficulty Describing Feelings; SSO, Support from Significant others; SQ, Subjective sleep quality; PHQ-9, Patient Health Questionnaire-9; GAD-7, Generalized Anxiety Disorder-7.

This nomogram can be used to estimate the risk of NSSI in adolescents with depression. The practical steps for its application are as follows: First, locate the specific value of each variable for the individual on its corresponding scale axis, then project it upward to the “Points” axis to obtain the individual score points for that variable. Next, sum the individual score points of all variables to calculate the “Total Points.” Finally, locate this total score on the “Total Points” axis and project it vertically downward to the “NSSI risk” axis to estimate the individual’s probability of NSSI.

### Nomogram model performance assessment

To comprehensively evaluate the performance and stability of the nomogram, an assessment framework was established across three core dimensions: discriminative ability, calibration, and clinical utility. The area under the ROC (AUC) was 0.754 (95% CI: 0.726-0.781) in the training set and AUC = 0.748 (95% CI: 0.707-0.789) in the validation set, indicating reliable discriminative performance. Results from the Hosmer-Lemeshow goodness-of-fit test indicated a good fit for the nomogram model in both datasets (training set: *χ²* = 14.40, *p* = 0.072; validation set: *χ²* = 9.81, *p* = 0.278) (**Supplementary Figure 1**). The DCA curves suggested the satisfactory clinical usefulness of our model (**Supplementary Figure 2**). DCA curves showed that, in the validation set, the nomogram provided a higher net benefit than both the treat-all and treat-none strategies across a main threshold probability range of 0.4–0.8. Given the relatively high prevalence of NSSI in this clinical sample, the advantage of the nomogram became more apparent at moderate-to-high thresholds.

## Discussion

Using random forest for variable selection and a nomogram for probability estimation, this study identified multiple factors associated with NSSI among adolescents with depression, including depression severity, use of sleeping medication, lower perceived family support, difficulty identifying emotions, externally oriented thinking, younger age, female sex and hallucination.

At the individual level, consistent with previous studies, depression (16, 17), female (48) and younger age (15) emerged as important factors associated with NSSI. Specifically, a PHQ-9 score of 16 or higher was associated with a marked increase in the risk of NSSI. The occurrence of NSSI among adolescents with MDD has been linked to the synergistic interaction of multiple mechanisms, including psychosocial factors, hypothalamic-pituitary-adrenal (HPA) axis function, and pain perception (49). From a psychosocial perspective, adverse childhood experiences (ACEs) represent a key risk factor. Adolescents with NSSI who report more severe ACEs exhibit significantly elevated hair cortisol levels (50), which is closely related to HPA axis dysfunction and epigenetic alterations commonly observed in individuals with comorbid MDD and NSSI (51). Moreover, psychosocial environments may be associated with the onset of NSSI by modulating gene expression (52). Physiologically, adolescents with NSSI demonstrate higher thresholds and tolerance to thermal and cold pain compared with healthy controls (53), providing a physiological basis for NSSI. Functionally, NSSI may rapidly reduce the intensity of negative affect and divert attention away from distressing ruminative thoughts, thereby serving as a coping strategy for emotion regulation (54). Together, these mechanisms are interrelated and may be jointly associated with the occurrence of NSSI.

NSSI typically begins in early adolescence (16). This study further found that NSSI gradually declines after the age of 15. This may be a related to the immature emotional regulation abilities of adolescents before 15. When confronted with stress or negative life events, limited emotional management skills, insufficient cognitive coping may be associated with engagement in NSSI as a proposed means of emotion regulation (55). The prevalence of NSSI is higher among female adolescents. This may be related to a greater tendency for females to internalize emotional distress, increasing the likelihood of using NSSI as an emotion regulation strategy, whereas males are more likely to exhibit externalizing behaviors such as aggression and impulsivity (56).

Our study identified a significant association between sleep medication use and NSSI. However, this association should be interpreted carefully. First, this study did not include detailed classification of medications, participants may have categorized various nighttime medications (including benzodiazepines, antidepressants, etc.) under the label of hypnotics. Such imprecise categorization may limit the accurate interpretation of the association between medication use and NSSI. Second, prior studies have reported conflicting findings on the link between antidepressants (including SSRIs) and suicide-related risks in adolescents. Pharmacoepidemiological studies have indicated a negative correlation between antidepressant utilization rates and suicide rates among children and adolescents (57). In contrast, meta-analyses of randomized controlled trials have suggested an increased risk of suicidal ideation or behaviors after medication in this population. Compared with placebo, antidepressant use was associated with 7–20 additional suicide-related adverse events per 1, 000 users (58). In addition, the association between antidepressants and suicide-related risks is complex. Depression represents a strong risk-related factor for self-injury and suicide in adolescents (59). Moreover, the emergence of suicide-related behaviors often prompts clinicians to initiate antidepressant treatment (60). As a result, it is difficult in clinical practice to distinguish whether suicide-related risks reflect new-onset risks attributable to pharmacotherapy or pre-existing risks that persist during treatment.

Regarding psychiatric symptoms, this study found that hallucinations were significantly associated with NSSI, which is consistent with previous findings on the relationship between auditory hallucinations (AHs) and self-injurious behavior (SIB) in adolescents (61). Previous studies suggest that there may be a bidirectional association between AHs and SIB in adolescents. AHs may be associated with subsequent SIB, while SIB may also reflect ongoing psychological distress and be associated with later AHs. Within-person analyses further indicate that this relationship may vary across developmental stages.

From a clinical perspective, AHs are often associated with psychosocial difficulties such as loneliness (62), social isolation (63), hopelessness (64), and emotion regulation difficulties (28). Therefore, auditory hallucinations may reflect not only psychotic experiences themselves, but also a state of heightened subjective distress, social disconnection, and emotional dysregulation. For adolescents with depression, recurrent or distressing auditory hallucinations may be accompanied by self-negation, hopelessness, and a perceived loss of control. In this context, NSSI may function as a maladaptive strategy for temporarily relieving internal distress or restoring a sense of control in some adolescents. From this perspective, hallucination-related symptoms may serve as an important marker of clinical complexity in the assessment of NSSI among adolescents with depression. In addition to assessing depression severity, clinicians should further evaluate the content, frequency, and distress associated with auditory hallucinations, as well as whether they are command-based or self-deprecating, to better understand the risk-related features of NSSI.

Among the dimensions of alexithymia, our findings indicate that difficulty identifying feelings is significantly associated with NSSI, which is consistent with evidence from meta-analyses (19, 20). Notably, difficulty identifying feelings is generally regarded as the core deficit of alexithymia. Neurobiological studies indicate that individuals with alexithymia show reduced activation in the right anterior insula and the anterior cingulate cortex, two regions involved in integrating emotional meaning, and dysfunction in these regions is associated with difficulties in identifying emotions (65). In addition, difficulty identifying feelings may impair social functioning. For example, it may be associated with poorer interpretation of others’ emotions during interpersonal conflicts, which in turn is related to greater relational tension (66). Taken together, these findings suggest that difficulty identifying feelings may be associated with NSSI through impaired emotional recognition and social adaptation in adolescents.

In contrast, the role of externally oriented thinking appears to be more complex. In the present study, externally oriented thinking was also significantly associated with NSSI. Although meta-analyses do not support a direct association between externally oriented thinking and NSSI, accumulating evidence suggests that externally oriented thinking may be associated with NSSI through indirect mechanisms. Empirical studies have reported that individuals with NSSI demonstrate longer pain detection latencies, reduced pain perception intensity, and prolonged pain tolerance during the cold pressor task (67), alterations that may be related to an externally oriented cognitive style. Such deficits may limit the ability to regulate intense emotions through interoceptive signals, impair accurate representation of emotion-related physiological states, and disrupt adaptive emotional responses. Ultimately, these deficits may be linked to greater reliance on maladaptive behaviors such as NSSI (68). Consequently, externally oriented thinking may function as a mediating mechanism through which alexithymia is linked to NSSI, rather than operating as an independent risk pathway. Moreover, among adolescents with depression, an externally focused cognitive style may further suppress attention to internal emotional states, exacerbating emotion regulation deficits, which represent a core correlate of NSSI.

Among different forms of social support, family support appears to exert the most prominent protective effect against NSSI. Specifically, higher levels of perceived family support are associated with a lower probability of current NSSI, which is consistent with previous studies (17, 26, 69, 70). As the system with which adolescents maintain the closest daily emotional bonds, families provide emotional acceptance and problem-solving support that may directly be linked to reduced impact of negative emotions on NSSI. One study focusing on hospitalized adolescents reported that support from authoritative figures (family members and medical staff) was closely associated with NSSI (69). However, the sample in that study consisted of inpatients whose autonomy was restricted in the hospital setting. In inpatient settings, the authority of medical staff is often linked to practical considerations such as discharge or referral, making such support more instrumental in nature. In contrast, the present study focuses on adolescents’ daily environments, where family support primarily provides emotional security. This non-utilitarian form of support may yield a more stable and enduring protective effect on emotion regulation. Additionally, longitudinal studies have found that teacher support is the only factor significantly and directly associated with subsequent NSSI (70), which differs from the findings of the present study. This discrepancy may be explained by two factors. First, the current study did not further differentiate subtypes of social support. Second, differences in study design and developmental stage may play a role: whereas the current cross-sectional study captures the contemporaneous association between family support and current NSSI, longitudinal designs are better positioned to detect the gradual, long-term influence of teacher support on behavioral development as adolescents mature.

The variable importance ranking derived from the ML model indicates that emotional abuse, emotional neglect, and verbal victimization were among the top-ranked variables associated with NSSI. Consistent with prior research (71, 72), these forms of ACEs exert comparably strong influences on NSSI and are therefore considered collectively in the following discussion. Accumulating evidence indicates that childhood emotional abuse and neglect exert sustained effects across development. Specifically, these experiences are associated with poorer emotion regulation capacities, show the strongest associations with adolescents’ self-injurious ideation (73), and increase the risk of NSSI regardless of the presence of comorbid depressive symptoms (72). Among adolescents with depression, childhood trauma is also directly associated with coping styles. To be specific, early adverse experiences directly drive patients to develop maladaptive coping patterns such as avoidance and aggression, and such behavioral responses induced by psychological stress are more prominent in individuals with MDD (74). From the perspective of brain development, childhood abuse may be associated with altered trajectories of brain development, and with differences in sensory systems as well as neural networks involved in threat detection, emotion regulation, and reward anticipation (75). In turn, subsequent alterations in emotional and reward systems may further potentiate the occurrence of NSSI (76). Random intercept cross-lagged panel model analyses show that, within individuals, baseline depressive symptoms predict increased subsequent recall of ACEs, whereas ACE recall did not predict later depression. These findings indicate that depression can reshape autobiographical memory of adversity, probably via negative emotional processing and memory bias (77).

In contrast, physical abuse did not show a statistically significant association with adolescent NSSI. This finding may be partly attributable to cultural factors. Within the context of traditional Chinese educational values, mild corporal punishment is sometimes perceived as a means of fostering resilience. Such cultural interpretations may attenuate individuals’ recognition of the potential harm associated with physical abuse, thereby weakening its observed association with NSSI (72).

This nomogram demonstrates potential clinical utility by integrating individual and psychosocial variables to estimate the probability of NSSI in clinical settings. It may provide a quantitative reference to support comprehensive psychosocial assessment and individualized clinical management. Nevertheless, several limitations should be acknowledged. First, the retrospective design precludes causal inference regarding the relationships between the associated variables and NSSI. Longitudinal studies are warranted to validate these associations and clarify their temporal dynamics. Second, all data were self-reported and lacked objective physiological measures such as blood biomarkers, which should be incorporated in future research. Third, although the sample was drawn from a nationwide multicenter cohort across 14 hospitals, it mainly consisted of treatment-seeking adolescents. This may result in an overrepresentation of moderate-to-severe NSSI cases, while underrepresenting community-based adolescents with mild or concealed self-injurious behaviors who do not seek medical care. Consequently, the generalizability of the model to the broader adolescent population requires further validation. Fourth, the study focused primarily on the independent effects of risk-related factors and did not fully explore potential interactions (e.g., the synergistic effect of alexithymia and childhood trauma). This may have led to an underestimation of the explanatory or classificatory value of combined clinical profiles. Fifth, detailed drug classification was not available in this study. Participants may have broadly classified various psychiatric medications taken at night as sleeping medication, which may limit the accurate interpretation of its association with NSSI. Due to the high event rate of NSSI among recruited clinical adolescents, this predictive model is primarily applicable for targeted risk stratification to prioritize high-risk individuals, instead of taking the place of standard clinical NSSI evaluation for all depressed adolescents. The complexity of machine learning may limit their interpretability and clinical applicability. Further development of explainable AI is needed to improve their practical use in clinical settings (36).

### Intervention implications for NSSI

The findings may have implications for clinical assessment and management of NSSI among adolescents with depression. First, clinicians should attach great importance to depressive symptoms and hallucinations among adolescents with NSSI. Concurrently, sleep problems should be incorporated into routine psychosocial and clinical assessments. Although alexithymia may hinder the effectiveness of psychotherapy, evidence suggests that targeted interventions can alleviate these traits (78). For adolescents with higher levels of alexithymia, emotion awareness training may be particularly beneficial. As a core component of Dialectical Behavior Therapy (DBT), this approach helps enhance the ability to identify and express emotions (79).

In clinical practice, enhancing family support should be prioritized. Parental training may help parents attend to adolescents’ emotional expression and enhance emotional support, rather than focusing solely on problem solving. Schools can play a complementary role by establishing effective home–school communication platforms, with educators paying particular attention to adolescents who lack sufficient family support and proactively offering assistance to strengthen the overall support system. Accordingly, schools and communities should strengthen protective measures for adolescents, reduce bullying and abuse, and enhance social support networks. In summary, interventions for adolescent NSSI should focus on improving family functioning and peer relationships, strengthening emotion regulation capacities, and paying particular attention to younger female adolescents.

## Conclusion

Based on random forest and logistic regression, the present study constructed and internally validated a nomogram to estimate the probability of NSSI among adolescents with depression. The model integrated multidimensional clinical and psychosocial variables at the individual, family, and school levels. Several variables associated with current NSSI were retained in the final model, including a depression, sleep medication use, difficulty identifying feelings, externally oriented thinking, younger age, insufficient perceived family support, female and hallucination. The nomogram demonstrated acceptable discriminative performance and calibration, suggesting that it may serve as a quantitative reference for clinical assessment and individualized clinical management in psychiatric outpatient services and adolescent mental health clinics.

## Data Availability

The datasets presented in this article are not readily available because they are restricted by ethical and privacy guidelines for adolescent mental health research. Requests to access the datasets should be directed to KZ email: cocozk1986@163.com.
