# Supplementary material for: Machine learning-based nomogram for non-suicidal self-injury among depressed adolescents: a multicentre study
Source: Front Psychiatry. 2026 Jul 17;17:1787248. doi: 10.3389/fpsyt.2026.1787248 (PMC13423768; doi:10.3389/fpsyt.2026.1787248)
Supplement: Supplementary file 1 [file DataSheet1.pdf]

Machine Learning-Based Nomogram for Non-Suicidal Self-Injury Among Depressed Adolescents: A Multicentre Study

Table S1. Multicollinearity diagnosis.

Variable	VIF
Age	3.899
PHQ	3.896
EY	3.896
GAD	3.118
DIF	2.874
SFR	2.463
SFA	2.459
SSO	2.395
DDF	2.313
EN	2.308
EA	2.128
VV	2.105
EM	2.010
SM	2.006
AP	1.969
EF	1.931
SQ	1.903
DY	1.834
SDI	1.761
PV	1.746
PN	1.676
SI	1.618
PB	1.612
SD	1.568
Hallucination	1.489
Delusion	1.447
HSE	1.396
Location	1.313
SA	1.161
EOT	1.147
USM	1.135
Gender	1.108
AHI	1.073
FHMD	1.023
PD	1.016

Abbreviation: USM: Use of sleeping medication; DIF: Difficulty Identifying Feelings; SFA: Support from Family; EY: Education years; EOT: Externally Oriented Thinking; VV: Verbal victimization; EN: Emotional neglect; SD: Sleep duration; AHI: Annual household income; EA: Emotional abuse; DDF: Difficulty Describing Feelings; SSO: Support from Significant others; SM: Social manipulation; AP: Attacks on property; SI: Sleep latency; PV: Physical victimization

Table S2. Comparison of characteristics between adolescents in the training and validation set.

Variable	Training Set (n=1641, 70%)	Validation set (n = 702, 30%)	*t/χ2*	*p*
FHMD (n, %)				
No	1492 (0.91)	629 (0.90)	0.85	0.36
Yes	149 (0.09)	73 (0.10)		
Age (mean, SD)	15.03 (1.64)	14.90 (1.68)	1.67	0.10
Gender (n, %)				
Boy	360 (0.22)	157 (0.22)	0.03	0.86
Girl	1281 (0.78)	545 (0.78)		
EY (mean, SD)	9.19 (1.73)	9.09 (1.77)	1.29	0.20
Location (n, %)				
Rural	1103 (0.67)	477 (0.68)	0.09	0.76
City	538 (0.33)	225 (0.32)		
AHI (n, %)				
≤ 20,000	245 (0.15)	100 (0.14)		
20,000 - 50,000	189 (0.12)	80 (0.11)	0.36	0.99
50,000 - 100,000	282 (0.17)	126 (0.18)		
100,000 - 500,000	395 (0.24)	167 (0.24)		
> 500,000	530 0.32 ()	229 (0.33)		
EF (n, %)				
≤6 years	209 (0.13)	92 (0.13)	0.11	0.99
7-9 years	617 (0.38)	265 (0.38)		
10-12 years	369 (0.22)	158 (0.23)		
≥13 years	446 (0.27)	187 (0.27)		
EM (n, %)				
≤6 years	365 (0.22)	167 (0.24)		
7-9 years	555 (0.34)	254 (0.36)	5.41	0.14
10-12 years	337 (0.21)	116 (0.17)		
≥13 years	384 (0.23)	165 (0.24)		
PD (n, %)				
No	51 (0.03)	14 (0.02)	1.87	0.17
Yes	1590 (0.97)	688 (0.98)		
Hallucination (n, %)				
None	1021 (0.62)	450 (0.64)	0.79	0.67
Possible	484 (0.29)	195 (0.28)		
Confirmed	136 (0.08)	57 (0.08)		
Delusion (n, %)				
None	1030 (0.63)	442 (0.63)		
Possible	331 (0.20)	154 (0.22)	1.89	0.39
Confirmed	280 (0.17)	106 (0.15)		
MPSS (mean, SD)				
SFA	14.99 (6.16)	15.35 (6.10)	-1.30	0.19
SFR	16.07 (6.84)	16.15 (6.76)	-0.24	0.81

SSO	15.85 (7.13)	15.92 (7.11)	-0.24	0.81
TAS (mean, SD)				
DIF	26.31 (6.10)	26.29 (5.89)	0.06	0.95
DDF	18.23 (3.54)	18.33 (3.48)	-0.60	0.55
EOT	23.30 (3.78)	23.23 (3.69)	0.42	0.68
CTQ (mean, SD)				
PB	7.25 (3.57)	7.07 (3.31)	1.13	0.26
PN	10.37 (3.34)	10.34 (3.32)	0.19	0.85
EN	16.03 (5.04)	16.01 (5.05)	0.09	0.93
EA	11.15 (4.76)	11.07 (4.67)	0.38	0.71
SA	5.88 (2.40)	5.88 (2.44)	-0.04	0.97
Bully (mean, SD)				
PV	1.92 (2.54)	2.00 (2.64)	-0.67	0.51
SM	3.46 (3.20)	3.32 (3.17)	0.98	0.33
VV	3.67 (3.03)	3.58 (2.99)	0.66	0.51
AP	2.34 (2.70)	2.35 (2.67)	-0.06	0.95
PSQI (mean, SD)				
SQ	1.70 (0.82)	1.68 (0.84)	0.53	0.60
SI	1.89 (1.04)	1.88 (1.01)	0.22	0.83
SD	1.23 (1.06)	1.23 (1.08)	0.11	0.92
HSE	0.96 (1.06)	1.01 (1.05)	-1.05	0.29
SDI	1.51 (0.72)	1.50 (0.73)	0.31	0.76
USM	1.08 (1.32)	1.05 (1.30)	0.51	0.61
DY	2.46 (0.82)	2.45 (0.82)	0.27	0.79
Depression (mean, SD)	16.90 (7.24)	16.76 (7.00)	0.43	0.67
Anxiety (mean, SD)	12.11 (6.34)	12.08 (6.03)	0.11	0.91

Abbreviation: USM: Use of sleeping medication; DIF: Difficulty Identifying Feelings; SFA: Support from Family; EY: Education years; EOT: Externally Oriented Thinking; VV: Verbal victimization; EN: Emotional neglect; SD: Sleep duration; AHI: Annual household income; EA: Emotional abuse; DDF: Difficulty Describing Feelings; SSO: Support from Significant others; SM: Social manipulation; AP: Attacks on property; SI: Sleep latency; PV: Physical victimization

Table S3. Dataset variables and descriptions for NSSI risk assessment.

Variable name	Meaning of variable	Type of variable	Assignment description
NSSI	Non-Suicidal Self-Injury	Categorical variable	0=no-NSSI, 1=NSSI
FHMD	Family history of mental disorders	Categorical variable	0=no, 1=yes
Age	Age	Numerical variable	Is unassigned
Gender	Gender	Categorical variable	0=male, 1=female
Location	Living area	Categorical variable	0=Rural, 1=City
EY	Education years	Numerical variable	Is unassigned
AHI	Annual household income	Categorical variable	0=≤20,000;<br>1=20,000–50,000;<br>2=50,000–100,000;<br>3=100,000–500,000;<br>4=>500,000
EF	Father's education level	Categorical variable	0=≤6 years;<br>1=7–9 years;<br>2=10–12 years;<br>3=≥13 years
EM	Mother's education level	Categorical variable	0=≤6 years;<br>1=7–9 years;<br>2=10–12 years;<br>3=≥13 years
PD	Physical disease	Categorical variable	0=No; 1=Yes
Hallucination	Hallucination	Categorical variable	0=None;<br>1=Possible;<br>2=Confirmed
Delusion	Delusion	Categorical variable	0=None;<br>1=Possible;<br>2=Confirmed
SFA	Support from Family	Numerical variable	Is unassigned
SFR	Support from Friends	Numerical variable	Is unassigned
SSO	Support from Significant others	Numerical variable	Is unassigned

Variable name	Meaning of variable	Type of variable	Assignment description
DIF	Difficulty Identifying Feelings	Numerical variable	Is unassigned
DDF	Difficulty Describing Feelings	Numerical variable	Is unassigned
EOT	Externally Oriented Thinking	Numerical variable	Is unassigned
PB	Physical abuse	Numerical variable	Is unassigned
PN	Physical neglect	Numerical variable	Is unassigned
EN	Emotional neglect	Numerical variable	Is unassigned
EA	Emotional abuse	Numerical variable	Is unassigned
SA	Sexual abuse	Numerical variable	Is unassigned
PV	Physical victimization	Numerical variable	Is unassigned
SM	Social manipulation	Numerical variable	Is unassigned
VV	Verbal victimization	Numerical variable	Is unassigned
AP	Attacks on property	Numerical variable	Is unassigned
SQ	Subjective sleep quality	Numerical variable	Is unassigned
SI	Sleep latency	Numerical variable	Is unassigned
SD	Sleep duration	Numerical variable	Is unassigned
HSE	Habitual sleep efficiency	Numerical variable	Is unassigned
SDI	Sleep disturbances	Numerical variable	Is unassigned
USM	Use of sleeping medication	Numerical variable	Is unassigned
DY	Daytime dysfunction	Numerical variable	Is unassigned
PHQ	Depression	Numerical variable	Is unassigned
GAD	Anxiety	Numerical variable	Is unassigned

Table S4. Hyperparameter Search Space and Optimal Hyperparameters for Machine Learning Models.

Model	Hyperparameters (with final values)	Candidate values / search range
Decision tree	minsplit=20; minbucket=10; cp=0.01; maxdepth=5; threshold=0.5623	minsplit fixed at 20;minbucket fixed at 10; cp fixed at 0.01;maxdepth fixed at 5; threshold selected using the Youden index
Random Forest	ntree=1000; mtry=3; nodesize=3; maxnodes=20; sampsize=mild_balance; threshold=0.5445	ntree fixed at 1000;mtry=2,3,4,5,6,8,11; nodesize=1,3,5,10,15;maxnodes=10,20,30,50, none;sampsize=balanced,mild_balance,original_bootstrap;threshold selected from OOB probabilities
XGBoost	max_depth=3; eta=0.07; min_child_weight=1; subsample=0.8; colsample_bytree=1.0; gamma=1; scale_pos_weight=0.315; lambda=1; nrounds=48	max_depth=3,4,5,6;eta=0.03,0.05,0.07; min_child_weight=1,3,5;subsample=0.7,0.8,0.9;colsample_bytree=0.6,0.8,1.0; gamma=0,0.5,1; nrounds up to 1000
LASSO	alpha=1; lambda=0.0060; threshold=0.7917	alpha fixed at 1;lambda automatically generated by cv.glmnet; continuous predictors standardized; categorical predictors dummy-coded; threshold selected using Youden index
SVM	kernel=RBF; cost=1; gamma=0.01; standardization applied; dummy coding applied; threshold=0.794	RBF kernel; cost=0.1,1,10; gamma=0.001,0.01,0.1; continuous predictors standardized; categorical predictors dummy-coded
LightGBM	objective=binary; metric=auc; boosting=gbdt;learning_rate=0.05; num_leaves=31; max_depth=-1; min_data_in_leaf=20; feature_fraction=0.8; bagging_fraction=0.8; bagging_freq=1; lambda_l2=0; scale_pos_weight=0.315; best_iter=51; threshold=0.471	Most parameters fixed; num_boost_round up to 1000 with early stopping
KNN	k=15; standardization applied; dummy coding applied; threshold=0.700	k=3,5,7,9,11,15,21;continuous predictors standardized;categorical predictors dummy-coded
Naive Bayes	laplace=0; usekernel=FALSE; adjust=0.5; threshold=0.874	laplace=0,0.5,1;usekernel=FALSE,TRUE; adjust=0.5,1,1.5,2

Table S5. Threshold-dependent validation metrics of the random forest model

Threshold	TP	TN	FP	FN	AUC	Accuracy	Specificity	Balanced accuracy	Sensitivity	PPV	NPV	PR-AUC	Brier score
Youden threshold, 0.544	413	99	69	121	0.755	0.729	0.589	0.681	0.773	0.857	0.450	0.902	0.163

Notes: TP: True Positive, TN: True Negative, FP: False Positive, FN: False Negative, PPV: Positive Predictive Value, NPV: Negative Predictive Value, PR-AUC: Precision-Recall Area Under the Curve

Table S6. Performance of PHQ-9 and PHQ-8 random forest models

Model	Accuracy	AUC	Sensitivity	PPV	NPV	Brier score
PHQ-9 RF	0.729	0.755	0.773	0.857	0.450	0.163
PHQ-8 RF	0.708	0.775	0.713	0.864	0.471	0.179

Note: PPV: Positive Predictive Value, NPV: Negative Predictive Value, AUC: Area Under the Curve

Table S7. SHAP Importance in PHQ-9 and PHQ-8 random forest models

Predictor	PHQ-9 rank	PHQ-9 mean |SHAP|	PHQ-8 rank	PHQ-8 mean |SHAP|	Rank change	Top-20 overlap
PHQ / PHQ8	1	0.153	3	0.133	-2	Yes
USM	2	0.117	1	0.149	+1	Yes
DIF	3	0.091	2	0.139	+1	Yes
Age / age	4	0.076	5	0.108	-1	Yes
SFA	5	0.072	—	—	—	No
GAD	6	0.072	10	0.091	-4	Yes
Hallucination	7	0.067	7	0.102	0	Yes
EA	8	0.067	9	0.097	-1	Yes
EY	9	0.065	12	0.082	-3	Yes
VV	10	0.062	11	0.090	-1	Yes
EN	11	0.055	8	0.099	+3	Yes
EOT	12	0.053	13	0.079	-1	Yes
Gender	13	0.053	18	0.061	-5	Yes
DDF	14	0.046	6	0.106	+8	Yes
AP	15	0.042	—	—	—	No
SSO	16	0.041	15	0.071	+1	Yes
AHI	17	0.037	—	—	—	No
PN	18	0.036	20	0.057	-2	Yes
SM	19	0.036	14	0.072	+5	Yes
SI	20	0.034	—	—	—	No
SDA	—	—	4	0.116	—	No
SD	—	—	16	0.062	—	No
Delusion	—	—	17	0.062	—	No
PV	—	—	19	0.058	—	No

Note. Rank change was calculated as PHQ-9 rank minus PHQ-8 rank; positive values indicate a higher ranking in the PHQ-8 model. Sixteen predictors overlapped between the two top-20 lists.

Table S8. Subgroup analysis of the random forest model in the validation cohort.

**Subgroup**	**N**	**Event_N**	**Accuracy**	**AUC**	**Sensitivity**	**PPV**	**Brier score**
Gender_Male	159	89	0.679	0.787	0.899	0.262	0.200
Gender_Female	544	419	0.778	0.726	0.981	0.709	0.161
Age < 15	279	222	0.806	0.794	0.982	0.764	0.133
Age ≥15	424	286	0.722	0.717	0.955	0.504	0.193

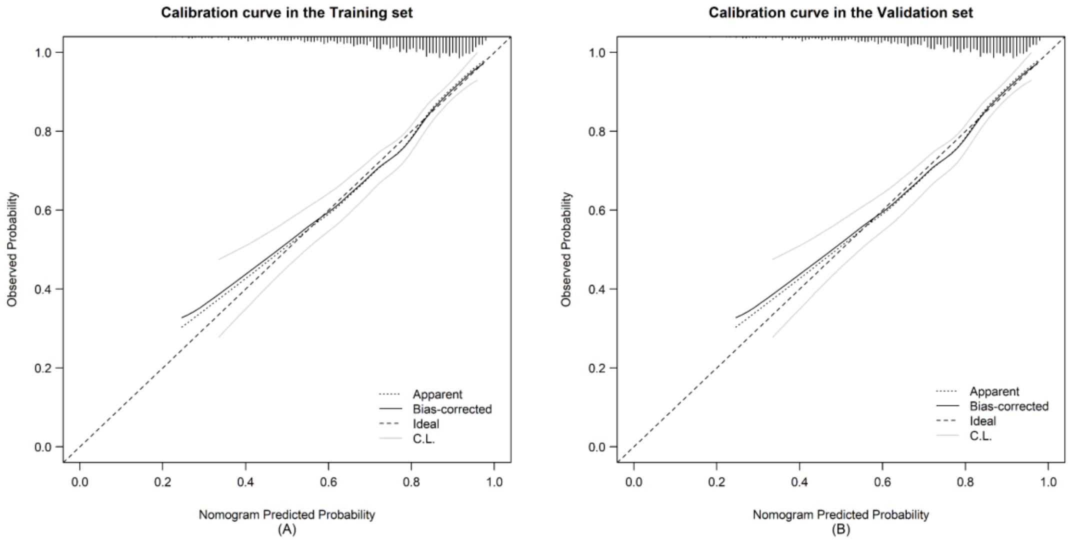

Fig. S1 Calibration curves of the nomogram model for NSSI. (A) for the training set and (B) for the validation set.

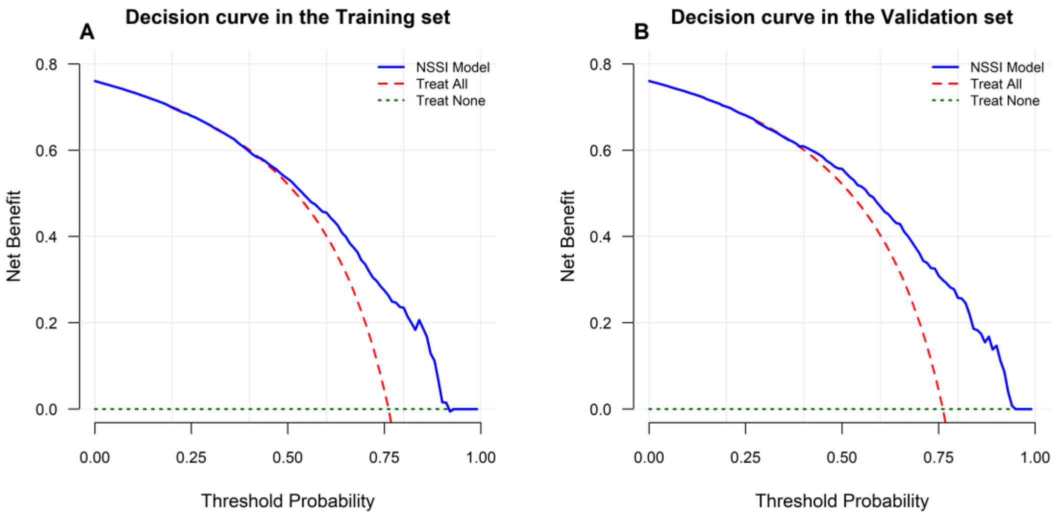

Fig. S2 Decision curve analysis (DCA) for the training set (A) and validation set (B), demonstrating the net clinical benefit of the nomogram model across different risk thresholds.

The "Nomogram model" curve represents the net benefit of using the proposed predictive model, while the "Treat All" and "Treat None" curves represent the strategies of treating all individuals as high risk or none as high risk, respectively. The higher net benefit of the "Nomogram model" across a wide range of risk thresholds suggests its clinical utility in identifying high-risk individuals more effectively than the alternative strategies.
